# Curiethérapie dans le traitement palliatif du cancer de l’œsophage

**DOI:** 10.11604/pamj.2015.20.59.5881

**Published:** 2015-01-22

**Authors:** Ahmedou Toulba, Hanae Bakkali, Salwa Boutayeb, Tayeb Kebdani, Samir Ahid, Noureddine Benjaafar

**Affiliations:** 1 Service de Radiothérapie, Institut Nation d'Oncologie, Faculté de médecine et Pharmacie de Rabat, Université Mohammed V Souissi, Rabat, Maroc; 2 Physique Médicale, Institut Nation d'Oncologie, Rabat, Maroc; 3 Equipe de Recherche Pharmaco-épidémiologie et Pharmaco-économie, Faculté de médecine et pharmacie de Rabat, Université Mohammed V Souissi, Rabat, Maroc; 4 Laboratoire de Biostatistique, de Recherche Clinque et d'Epidémiologie, Faculté de Médecine et Pharmacie de Rabat, Université Mohammed V Souissi, Rabat, Maroc

**Keywords:** Cancer de l′œsophage, dysphagie, curiethérapie HDR, Esophageal cancer, dysphagia, HDR brachytherapy

## Abstract

Les patients atteints du cancer de l’œsophage ont souvent une maladie localement avancée, la dysphagie est le symptôme majeur chez la plupart de ces patients, plusieurs modalités thérapeutiques ont été utilisées pour améliorer cette dysphagie. Le but de ce travail est d’étudier l'efficacité et la tolérance de la curiethérapie haut débit de dose (HDR) endo-luminale dans le traitement palliatif des cancers de l’œsophage inopérable. Sur une période de 15 ans, l’étude a inclus les patients atteints de cancer de l’œsophage inopérable et/ou métastatique avec une dysphagie, sans extension à l'hypopharynx ou a la trachée et qui ont bénéficié d'une curiethérapie HDR avec ou sans radiothérapie externe à visée palliative. Au total 46 patients ont été inclus dans l’étude, 58,7% étaient des hommes, 42,2% avaient une dysphagie grade 2 et 37,8% étaient aphagiques, 78,6% des patients étaient performance satus PS 2, l'amaigrissement à été trouvé chez 81,4%, la localisation de la tumeur était surtout au niveau du tiers moyen et inférieur dans 97,8%, la hauteur médiane de la tumeur était de 7 cm (5,5-9), le carcinome épidermoïde était le type histologique le plus fréquent chez 31 patients (70,5%). Après un médiane de suivi de 5 mois, l'amélioration de la dysphagie a été retrouvée chez 76% des malades (p1]. L′incidence la plus élevée est observée dans certains pays notamment en Asie et en Afrique, et l′incidence dans les pays développés occidentaux est en augmentation [2]. Selon le registre du cancer de Rabat 2006-2008, le cancer de l’œsophage est rare et constitue 1,5% de tous les cancers chez l'homme [3]. Le taux de survie globale à 5 ans est de 8%, avec 80% des décès liés à l’évolution locale de la maladie [4]. Pour la minorité des patients avec une maladie localisée, le traitement par radiochimiothérapie concomitante avec ou sans chirurgie permet une amélioration de la survie [5]. Plus de 50% des patients atteints de cancer de l′œsophage ont une maladie inopérable au moment du diagnostic due à une tumeur localement avancée, des métastases ou un mauvais état général avec une médiane de survie globale de 2,5 à 9,9 mois [6]. La majorité de ces patients ont besoin de soins palliatifs pour soulager la dysphagie qui est présente chez plus de 70% des patients et qui est responsable d'une dégradation importante de la qualité de vie [7]. À l′heure actuelle, plusieurs modalités de prise en charge sont disponibles pour le traitement palliatif de cette dysphagie. Les options de traitement les plus couramment utilisées comprennent le placement de stent métallique [8–10], le traitement au laser [11], et la curiethérapie avec ou sans radiothérapie externe et ou éventuellement une chimiothérapie [12–15]. Une méta-analyse a conclu qu'il n'y a pas de supériorité claire de l'une des méthodes utilisées dans la palliation du cancer de l’œsophage, le choix de l'une de ces méthodes dépend de l'expérience du centre et de l’état du patient. Cette méta-analyse a aussi démontré que la pose d'une prothèse métallique expansible et la curiethérapie étaient les deux méthodes de référence, la première étant la plus rapide pour améliorer la dysphagie, mais la seconde constitue une réelle alternative pouvant améliorer la survie et la qualité de vie [16]. Un inconvénient du traitement au laser est la nécessité de répéter les séances afin d'obtenir et de maintenir le bénéfice [17, 18]. Dans notre centre la curiethérapie est souvent utilisée pour pallier à la dysphagie. Le but de notre travail est de déterminer l'efficacité et la tolérance de la curiethérapie dans le traitement palliatif du cancer de l’œsophage.

## Introduction

Le cancer de l´œsophage est le sixième cancer chez l'homme et le neuvième chez la femme par ordre de fréquence et constitue la sixième cause de mortalité par cancer dans le monde [1]. L´incidence la plus élevée est observée dans certains pays notamment en Asie et en Afrique, et l´incidence dans les pays développés occidentaux est en augmentation [2]. Selon le registre du cancer de Rabat 2006-2008, le cancer de l’œsophage reste rare et constitue 1,5% de tous les cancers chez l'homme [3]. Le taux de survie globale à 5 ans est de 8%, avec 80% des décès liés à l’évolution locale de la maladie [4]. Pour la minorité des patients avec une maladie localisée, le traitement par radiochimiothérapie concomitante avec ou sans chirurgie permet une amélioration de la survie [5]. Plus de 50% des patients atteints de cancer de l´œsophage ont une maladie inopérable au moment du diagnostic due à une tumeur localement avancée, des métastases ou un mauvais état général avec une médiane de survie globale de 2,5 à 9,9 mois [6]. La majorité de ces patients ont besoin de soins palliatifs pour soulager la dysphagie qui est présente chez plus de 70% des patients et qui est responsable d'une dégradation importante de la qualité de vie [7]. À l´heure actuelle, plusieurs modalités de prise en charge sont disponibles pour le traitement palliatif de cette dysphagie. Les options de traitement les plus couramment utilisées comprennent le placement de stent métallique [8–10], le traitement au laser [11], et la curiethérapie avec ou sans radiothérapie externe et ou éventuellement une chimiothérapie [12–15]. Une méta-analyse a conclu qu'il n'y a pas de supériorité claire de l'une des méthodes utilisées dans la palliation du cancer de l’œsophage, le choix de l'une de ces méthodes dépend de l'expérience du centre et de l’état du patient. Cette méta-analyse a aussi démontré que la pose d'une prothèse métallique expansible et la curiethérapie étaient les deux méthodes de référence, la première étant la plus rapide pour améliorer la dysphagie, mais la seconde constitue une réelle alternative pouvant améliorer la survie et la qualité de vie [16]. Un inconvénient du traitement au laser est la nécessité de répéter les séances afin d'obtenir et de maintenir le bénéfice [17, 18]. Dans notre centre la curiethérapie est souvent utilisée pour pallier à la dysphagie. Le but de notre travail est de déterminer l'efficacité et la tolérance de la curiethérapie dans le traitement palliatif du cancer de l’œsophage.

## Méthodes

Il s'agit d'une étude rétrospective réalisée auprès des patients traités entre Janvier 1998 et Décembre 2012 au service de radiothérapie-curiethérapie à l'Institut National d'Oncologie (INO) de RABAT. Les patients inclus dans l’étude étaient ceux qui ont un cancer de l’œsophage ou la jonction gastro-œsophagienne inopérable et/ou métastatique et avec une dysphagie progressive grade 2-4 [15] et qui ont bénéficié d'un traitement à visée palliative par une curiethérapie endoluminale. Les patients présentant des tumeurs de l’œsophage avec extension à l'hypopharynx, fistule trachéo-oesophagienne ou avec extension trachéaleont été exclus de l’étude. L’évaluation de l'efficacité du traitement reposait principalement sur l'amélioration de la dysphagie qui est définie par la diminution d'au moins un grade à un mois après la fin de curiethérapie [13]. La tolérance est évaluée par la survenue des complications. Les données recueillies pour chaque patient étaient sociodémographiques (l’âge et le sexe), cliniques (le grade de la dysphagie, l’état général, l'amaigrissement, le type histologique, la localisation de la tumeur, sa hauteur et le stade clinique de la maladie), thérapeutiques (la dose de la radiothérapie, le fractionnement, l’étalement, le délai entre la radiothérapie et la curiethérapie et le protocole de la curiethérapie) et évolutives (l'amélioration de la dysphagie, la prise du poids et la survenue des complications). L'irradiation externe a été réalisée à l'aide d'un accélérateur linéaire délivrant des photons X de 25 MV ou des rayons gamma du Cobalt 60. La dose était de 30 Gray en 10 fractions avec une médiane d’étalement de 15 jours [13–16, 5]. La curiethérapie a été réalisée à l'aide de l'applicateur œsophagien chez Nucletron de diamètre variable de 4 à 8 mm qui constitue un vecteur pour la source radioactive. Le manchon externe de l'applicateur permet de réduire la dose au niveau de la muqueuse œsophagienne.

La zone tumorale a été repérée à l'aide des données endoscopiques et radiologiques (transit oeso-gastro-duodénal et scanner cervico-thoracique). La mise en place de l'applicateur a été réalisée sous anesthésie locale et elle est similaire à la mise en place d'une sonde gastrique. La fixation a été faite à la bouche à l'aide d'un cordon. Le contrôle de l'application a été fait par une radiographie de face après insertion d'une source fictive radio-opaque à l'intérieur de l'applicateur. Ce contrôle radiologique permet de tracer le volume cible tumoral et de repérer la première position et la dernière position de la source fictive pour réaliser la dosimétrie. Le volume cible tumoral comprend la hauteur tumorale macroscopique initiale avec une marge de sécurité de 2 cm en amont et en aval. La dose est prescrite à 0,5 cm de la surface de l'applicateur de part et d'autre, soit un manchon de 1 cm. Le calcul de la distribution de dose permet d'avoir une isodose de référence (100%) engendrée par les différentes positions d'arrêts et leurs durées d'arrêts. Après la validation de la dosimétrie, le plan de traitement est envoyé par réseau à l'unité de commande et de contrôle ou l'applicateur est lié au projecteur de source d'Iridium 192 pour l'exécution de l'irradiation. Les données ont été saisies et analysées à l'aide du logiciel SPSS13.0; les variables qualitatives ont été exprimées en effectif et en%, et les variables quantitatives en moyenne et écart type ou en médiane et quartiles. L’étude analytique a été faite avec le test d'homogénéité marginale, Khi-deux ou test exact de Fisher. L'analyse de la survie a été faite par la méthode Kaplan-Meier. Le seuil de signification p a été fixé à 0.05.

## Résultats

**Figure 1:**
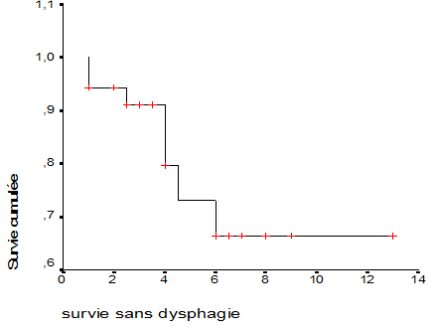
Courbe de survie sans dysphagie

Au terme de notre étude 46 patients ont été collectés, le sexe ratio H/F était de 1.4, l’âge moyen était de 62.4 ± 13.3 ans. Dix neuf patients (42.2%) avaient une dysphagie grade 2 et 17 autres (37.8%) étaient aphagiques (grade 4). Trente trois patients étaient classés PS 2. L'amaigrissement a été noté chez 35 patients (81.4%). La localisation était le tiers inférieur chez 23 (51.1%) patients et le tiers moyen chez 21 (46.7%) patients. La hauteur médiane de la tumeur était 7cm (5,5-9). Le type histologique le plus fréquent était le carcinome épidermoide chez 70.5% des patients (Tableau 1). Tous les malades ont eu une curiethérapie HDR, 35 patients ont bénéficié d'une radiothérapie externe avant celle-ci. Le délai médian entre la radiothérapie externe et la curiethérapie était de 21.5 jours (15-34.75). La dose délivrée par fraction variait entre 5 et 10 Gray, 85% des patients ont reçu une dose de 7 Gray par fraction en 1 à 4 séances. Les séances ont été réalisées avec une semaine d'intervalle. Au total 117 applications de curiethérapie HDR ont était réalisées chez ces patients. Les malades ont été revus à un mois après la fin du traitement puis chaque trois mois. Après une médiane de suivi de 5 mois, l'amélioration de la dysphagie a été constatée chez 35 patients (76%) avec P< 0.001 (Tableau 2). La médiane de la survie sans dysphagie était de 4 mois avec un intervalle de confiance (IC) 95%; IC 95% (3,185 - 4,815) (Figure 1). L'association de la radiothérapie à la curiethérapie n'améliore pas la dysphagie avec P à 0.9, la curiethérapie en une ou deux fractions versus plus de deux fractions ne montre pas de différence en terme de soulagement de la dysphagie (p à 0.9). Concernant les complications, 4 patients (8.7%) ont présenté une sténose œsophagienne et 3 (6.5%) autres une fistule trachéo-œsophagienne.

**Tableau 1:** Caractéristiques des patients

Caractéristiques	N = 46
**Sexe masculin**	27 (58.7%)
**Age**	
<50 ans	8 (17.4%)
51-70 ans	26 (56.5%)
>71 ans	12 (26.1%)
**Dysphagie**	
Grade 2	19 (42.2%)
Grade 3	9 (20%)
Grade 4	17 (37.8%)
**Performance Statut (OMS)**	
2	33 (78.6%)
3	9 (21.4%)
**Amaigrissement**	35 (81.4%)
**Localisation**	
Tiers supérieur	1 (2.2%)
Tiers moyen	21 (46.7%)
Tiers inferieur	23 (51.1%)
Hauteur de la tumeur *	7 (5,5-9)
**Type histologique**	
Carcinome épidermoîde	31 (70.5%)
Adénocarcinome	13 (29.5%)

variable exprimée en médiane (quartiles)

**Tableau 2:** Grade de dysphagie avant et après la curiethérapie

	Grade de dysphagie à l'admission n(%)	Grade de dysphagie après la curiethérapie après une médiane de suivi de 5 mois n(%)	P <0.001
Garde 0	0	17 (37%)	
Grade 1	0	14 (30.4%)	
Grade 2	19 (42.2%)	6 (13%)	
Grade 3	9 (20%)	6 (13%)	
Grade 4	17 (37.8%)	3 (6.5%)	

## Discussion

Il s'agit de la première étude réalisée au Maroc qui montre l'apport de la curiethérapie dans la palliation de la dysphagie. Notre étude a montré une efficacité chez les trois quarts des patients avec une toxicité acceptable. Le cancer de l’œsophage est associé à un pronostic péjoratif dû au diagnostic qui est souvent tardif limitant la possibilité d'un traitement à visée curative. La dysphagie constitue le symptôme majeur responsable d'une diminution de la qualité de vie et d'une dégradation de l’état général secondaire à la dénutrition qui va aggraver le pronostic de la maladie déjà sombre. Plusieurs publications ont étudié la place de la curiethérapie endoluminale de haut débit de dose dans le traitement palliatif de la dysphagie chez les malades atteints d'un cancer de l’œsophage localement avancé [13, 19–21]. Ces études ont montré que la curiethérapie endoluminale permet une amélioration de la dysphagie chez 50 à 80% des cas. Dans notre étude l'amélioration a été trouvée chez 76% des cas. Un essai publié récemment a randomisé 219 patients, il a monté que l'association de la radiothérapie externe à la curiethérapie améliore la survie sans dysphagie de 36 jours (200 vs 164 jours) avec aussi une amélioration significative du score de la dysphagie, de l'odynophagie, les régurgitations, les douleurs thoraciques et du performance status (PS), par contre il n'y avait pas de différence entre la prise du poids, la toxicité et la survie globale [13]. Un autre essai randomisé qui a comparé la curiethérapie au traitement combiné radiothérapie externe et curiethérapie, n'a pas montré une amélioration de la survie sans dysphagie quand on ajoute une curiethérapie [15]. Dans notre série le traitement combiné n'est pas associé à une amélioration significative de la dysphagie mais l'effectif était faible pour conclure.

Plusieurs essais ont comparé la curiethérapie en une seule fraction, deux fractions ou multiples. Un essai de l'l'International Atomic Energy Agency (IAEA) qui a randomisé 232 patients atteints d'un carcinome épidermoïde de l’œsophage inopérable entre curiethérapie HDR 18 Gy en 3 fractions et 16 Gy en deux fractions n'a pas démontré une différence entre ces deux schémas en terme de survie sans dysphagie, survie globale et la survenue de complications (fistules et sténoses), une amélioration de la dysphagie dans 80% des cas et une durée médiane de la palliation de 214 jours a été retrouvée. En analyse multivariée, seuls le sexe et l'indice de l’état général étaient les facteurs corrélés à la survie sans récidive [22]. D'autres auteurs ont utilisé une dose de curiethérapie de 12Gy en une seule fraction [8]. Malgré l'absence de différence entre traitement monofractionné et en multiples fractions, l'habitude de notre centre est de choisir le traitement fractionné et dans notre série le schéma avec multiples fractions est le plus souvent utilisé, seulement 5 patients ont bénéfice d'une seule fraction de 10 Gy. Les complications les plus couramment retrouvées sont les fistules, les sténoses, les ulcérations, les perforations et les hémorragies avec des taux entre 10 et 30% selon les études [23, 24]. Dans une série publiée les taux complications étaient de 30%, mais 36% de ces patients ont reçu une curiethérapie pour une dysphagie récidivante prétraitée ce qui peut majorer les complications [25]. Dans notre étude le taux de complications était de 15.2%. Dans notre étude, les données concernant la prise du poids et le recours à un autre traitement de la dysphagie n’étaient pas exploitées dans le dossier clinque des patients.

## Conclusion

La curiethérapie HDR endoluminale de l’œsophage constitue un moyen d'efficacité et de toxicité acceptable dans la palliation de la dysphagie chez les patients atteints du cancer de l’œsophage avancé non éligible à un traitement curatif.
